# Supplementary material for: Hot spot prediction in protein-protein interactions by an ensemble system
Source: BMC Syst Biol. 2018 Dec 31;12(Suppl 9):132. doi: 10.1186/s12918-018-0665-8 (PMC6311905; doi:10.1186/s12918-018-0665-8)
Supplement: Supplementary file 2 — All relevant MATLAB code. A simple Matlab implement of our predictor. (ZIP 19,742 kb) [file 12918_2018_665_MOESM2_ESM.zip › Additional file 2/Read ASA/ASA Property/ASEdb/3HHRB.txt]

 NetSurfP - Protein Surface Accessibility and Secondary Structure Predictions

  

|  |  |  |
| --- | --- | --- |
|  |  | NetSurfP - Protein Surface Accessibility and Secondary Structure Predictions  Technical University of Denmark |

  


---

```
# For publication of results, please cite:
# A generic method for assignment of reliability scores applied to solvent accessibility predictions.
# Bent Petersen, Thomas Nordahl Petersen, Pernille Andersen, Morten Nielsen and Claus Lundegaard
# BMC Structural Biology 2009, 9:51 doi:10.1186/1472-6807-9-51
#
# Column 1: Class assignment - B for buried or E for Exposed - Threshold: 25% exposure, but not based on RSA
# Column 2: Amino acid
# Column 3: Sequence name
# Column 4: Amino acid number
# Column 5: Relative Surface Accessibility - RSA
# Column 6: Absolute Surface Accessibility
# Column 7: Z-fit score for RSA prediction
# Column 8: Probability for Alpha-Helix
# Column 9: Probability for Beta-strand
# Column 10: Probability for Coil
E E  3HHRB                  1    0.787 137.419   1.793   0.003   0.003   0.994
B P  3HHRB                  2    0.188  26.734  -2.853   0.005   0.015   0.979
E K  3HHRB                  3    0.573 117.825   0.675   0.005   0.262   0.733
E F  3HHRB                  4    0.414  83.090  -0.394   0.004   0.514   0.481
B T  3HHRB                  5    0.105  14.522   0.330   0.004   0.616   0.381
E K  3HHRB                  6    0.322  66.256   0.676   0.001   0.900   0.099
B C  3HHRB                  7    0.041   5.742  -0.182   0.001   0.959   0.040
B R  3HHRB                  8    0.239  54.754   1.076   0.001   0.959   0.040
B S  3HHRB                  9    0.131  15.377  -0.714   0.001   0.900   0.099
E P  3HHRB                 10    0.404  57.328   0.575   0.004   0.514   0.481
E E  3HHRB                 11    0.535  93.430  -0.114   0.005   0.045   0.951
E R  3HHRB                 12    0.361  82.577   0.334   0.005   0.045   0.951
E E  3HHRB                 13    0.409  71.400   0.075   0.004   0.420   0.576
E T  3HHRB                 14    0.264  36.672   0.957   0.002   0.816   0.182
B F  3HHRB                 15    0.020   4.114   1.043   0.001   0.900   0.099
B S  3HHRB                 16    0.183  21.448   1.246   0.001   0.959   0.040
B C  3HHRB                 17    0.014   2.008   1.507   0.001   0.959   0.040
B H  3HHRB                 18    0.183  33.306   1.004   0.001   0.959   0.040
B W  3HHRB                 19    0.046  11.087  -0.334   0.001   0.900   0.099
E T  3HHRB                 20    0.409  56.770   0.835   0.003   0.718   0.279
B D  3HHRB                 21    0.255  36.717  -0.124   0.004   0.197   0.799
E E  3HHRB                 22    0.449  78.440  -0.893   0.005   0.045   0.951
E V  3HHRB                 23    0.580  89.115  -0.874   0.005   0.015   0.979
E H  3HHRB                 24    0.475  86.330  -2.102   0.018   0.019   0.964
E H  3HHRB                 25    0.539  98.080  -2.080   0.018   0.019   0.964
E G  3HHRB                 26    0.516  40.609  -2.544   0.005   0.015   0.979
B T  3HHRB                 27    0.239  33.094  -2.225   0.005   0.015   0.979
E K  3HHRB                 28    0.504 103.755  -1.789   0.005   0.045   0.951
E N  3HHRB                 29    0.460  67.285  -1.075   0.004   0.138   0.858
E L  3HHRB                 30    0.416  76.206  -1.270   0.005   0.336   0.660
B G  3HHRB                 31    0.231  18.164  -1.178   0.004   0.616   0.381
E P  3HHRB                 32    0.277  39.363  -0.443   0.002   0.816   0.182
B I  3HHRB                 33    0.067  12.451   0.314   0.001   0.959   0.040
B Q  3HHRB                 34    0.161  28.790   0.368   0.001   0.959   0.040
B L  3HHRB                 35    0.030   5.456   0.391   0.001   0.959   0.040
B F  3HHRB                 36    0.102  20.512   0.465   0.001   0.959   0.040
B Y  3HHRB                 37    0.064  13.655   0.425   0.001   0.959   0.040
B T  3HHRB                 38    0.148  20.528   0.178   0.001   0.959   0.040
E R  3HHRB                 39    0.275  62.883  -0.599   0.011   0.918   0.071
E R  3HHRB                 40    0.329  75.272  -0.637   0.022   0.552   0.426
E N  3HHRB                 41    0.422  61.825  -1.589   0.021   0.279   0.699
E T  3HHRB                 42    0.304  42.165  -1.200   0.019   0.141   0.840
E Q  3HHRB                 43    0.605 108.071  -1.001   0.019   0.141   0.840
E E  3HHRB                 44    0.604 105.589  -0.565   0.018   0.088   0.893
E W  3HHRB                 45    0.502 120.851  -0.678   0.018   0.088   0.893
E T  3HHRB                 46    0.640  88.782  -0.780   0.018   0.088   0.893
E Q  3HHRB                 47    0.620 110.714  -0.014   0.019   0.141   0.840
E E  3HHRB                 48    0.498  87.036   0.969   0.022   0.359   0.619
E W  3HHRB                 49    0.377  90.693  -0.069   0.021   0.451   0.528
E K  3HHRB                 50    0.409  84.152   1.158   0.021   0.451   0.528
E E  3HHRB                 51    0.565  98.688   0.669   0.022   0.359   0.619
B C  3HHRB                 52    0.071   9.954  -0.873   0.004   0.085   0.910
E P  3HHRB                 53    0.476  67.587  -0.977   0.004   0.085   0.910
E D  3HHRB                 54    0.579  83.492  -0.691   0.021   0.279   0.699
B Y  3HHRB                 55    0.272  58.084  -0.743   0.021   0.279   0.699
E V  3HHRB                 56    0.367  56.454  -0.694   0.004   0.420   0.576
E S  3HHRB                 57    0.582  68.246  -1.639   0.005   0.336   0.660
E A  3HHRB                 58    0.590  64.996  -2.852   0.004   0.138   0.858
E G  3HHRB                 59    0.387  30.481  -0.936   0.004   0.138   0.858
E E  3HHRB                 60    0.463  80.834  -0.694   0.005   0.336   0.660
B N  3HHRB                 61    0.235  34.433  -1.015   0.004   0.616   0.381
B S  3HHRB                 62    0.247  28.902  -0.003   0.002   0.816   0.182
B C  3HHRB                 63    0.027   3.847  -0.604   0.001   0.900   0.099
E Y  3HHRB                 64    0.266  56.930   0.498   0.001   0.900   0.099
B F  3HHRB                 65    0.040   8.068   0.572   0.002   0.816   0.182
B N  3HHRB                 66    0.246  35.941   0.038   0.004   0.616   0.381
E S  3HHRB                 67    0.298  34.972  -0.353   0.021   0.279   0.699
E S  3HHRB                 68    0.585  68.609  -1.643   0.019   0.141   0.840
E F  3HHRB                 69    0.534 107.134  -0.909   0.004   0.197   0.799
B T  3HHRB                 70    0.204  28.239  -1.036   0.004   0.197   0.799
E S  3HHRB                 71    0.432  50.595  -1.023   0.005   0.336   0.660
E I  3HHRB                 72    0.371  68.672  -1.074   0.004   0.420   0.576
E W  3HHRB                 73    0.373  89.803  -0.912   0.004   0.514   0.481
B I  3HHRB                 74    0.239  44.271  -0.192   0.003   0.718   0.279
E P  3HHRB                 75    0.369  52.333   0.412   0.001   0.900   0.099
B Y  3HHRB                 76    0.036   7.629   0.398   0.001   0.959   0.040
B C  3HHRB                 77    0.174  24.472   1.265   0.001   0.959   0.040
B I  3HHRB                 78    0.016   2.960   1.465   0.001   0.959   0.040
B K  3HHRB                 79    0.109  22.360   1.180   0.001   0.959   0.040
B L  3HHRB                 80    0.018   3.296   1.338   0.001   0.959   0.040
E T  3HHRB                 81    0.258  35.812   1.431   0.001   0.959   0.040
B S  3HHRB                 82    0.061   7.114  -0.827   0.001   0.900   0.099
E N  3HHRB                 83    0.387  56.671  -0.340   0.004   0.616   0.381
E G  3HHRB                 84    0.355  27.962  -0.865   0.004   0.197   0.799
E G  3HHRB                 85    0.527  41.443  -1.341   0.004   0.138   0.858
E T  3HHRB                 86    0.516  71.597  -1.546   0.004   0.197   0.799
E V  3HHRB                 87    0.330  50.660  -1.566   0.005   0.262   0.733
E D  3HHRB                 88    0.579  83.391  -0.749   0.004   0.197   0.799
E E  3HHRB                 89    0.457  79.873  -1.518   0.005   0.336   0.660
E K  3HHRB                 90    0.408  83.864  -0.119   0.004   0.616   0.381
E C  3HHRB                 91    0.420  59.024  -0.498   0.003   0.718   0.279
B F  3HHRB                 92    0.073  14.731  -0.160   0.004   0.616   0.381
E S  3HHRB                 93    0.445  52.189   0.770   0.022   0.552   0.426
B V  3HHRB                 94    0.179  27.528  -0.674   0.021   0.451   0.528
E D  3HHRB                 95    0.402  57.871  -0.791   0.022   0.359   0.619
E E  3HHRB                 96    0.405  70.788  -0.636   0.021   0.279   0.699
B I  3HHRB                 97    0.226  41.884  -0.082   0.021   0.451   0.528
B V  3HHRB                 98    0.097  14.878  -0.468   0.022   0.552   0.426
E Q  3HHRB                 99    0.345  61.599   0.924   0.021   0.451   0.528
B P  3HHRB                100    0.142  20.107  -0.874   0.004   0.197   0.799
E D  3HHRB                101    0.525  75.710  -0.387   0.005   0.045   0.951
B P  3HHRB                102    0.135  19.171  -0.103   0.018   0.019   0.964
B P  3HHRB                103    0.145  20.618  -1.401   0.018   0.019   0.964
E I  3HHRB                104    0.434  80.271  -1.094   0.018   0.088   0.893
E A  3HHRB                105    0.450  49.645  -0.825   0.019   0.141   0.840
B L  3HHRB                106    0.139  25.451   0.786   0.005   0.336   0.660
E N  3HHRB                107    0.525  76.860   0.026   0.004   0.514   0.481
B W  3HHRB                108    0.117  28.235  -0.497   0.003   0.718   0.279
E T  3HHRB                109    0.392  54.426   0.398   0.002   0.816   0.182
E L  3HHRB                110    0.354  64.854  -0.355   0.021   0.756   0.223
E L  3HHRB                111    0.359  65.678  -0.294   0.023   0.655   0.322
E N  3HHRB                112    0.547  80.066  -1.059   0.022   0.359   0.619
E V  3HHRB                113    0.443  68.120  -1.031   0.022   0.359   0.619
E S  3HHRB                114    0.494  57.920  -1.470   0.022   0.359   0.619
E L  3HHRB                115    0.350  64.158  -1.658   0.020   0.205   0.775
E T  3HHRB                116    0.543  75.259  -1.577   0.018   0.088   0.893
E G  3HHRB                117    0.595  46.819  -2.157   0.004   0.085   0.910
E I  3HHRB                118    0.382  70.615  -0.939   0.004   0.138   0.858
E H  3HHRB                119    0.453  82.473  -0.809   0.004   0.138   0.858
E A  3HHRB                120    0.305  33.655  -1.155   0.005   0.262   0.733
B D  3HHRB                121    0.251  36.212   0.142   0.003   0.718   0.279
B I  3HHRB                122    0.025   4.532   0.304   0.001   0.900   0.099
B Q  3HHRB                123    0.142  25.379   0.669   0.001   0.900   0.099
B V  3HHRB                124    0.018   2.767   1.310   0.001   0.900   0.099
B R  3HHRB                125    0.126  28.923   0.144   0.011   0.918   0.071
B W  3HHRB                126    0.036   8.682  -0.306   0.021   0.756   0.223
E E  3HHRB                127    0.411  71.854   0.530   0.004   0.514   0.481
E A  3HHRB                128    0.317  34.889  -0.132   0.004   0.138   0.858
B P  3HHRB                129    0.164  23.314  -1.215   0.018   0.047   0.935
E R  3HHRB                130    0.507 116.195  -1.794   0.018   0.047   0.935
E N  3HHRB                131    0.430  62.981  -2.058   0.018   0.047   0.935
E A  3HHRB                132    0.350  38.515  -1.521   0.018   0.047   0.935
E D  3HHRB                133    0.590  85.091  -2.158   0.018   0.047   0.935
E I  3HHRB                134    0.401  74.167  -1.827   0.018   0.047   0.935
E Q  3HHRB                135    0.568 101.498  -1.801   0.018   0.088   0.893
E K  3HHRB                136    0.539 110.913  -1.733   0.004   0.085   0.910
B G  3HHRB                137    0.274  21.580  -1.815   0.004   0.138   0.858
B W  3HHRB                138    0.289  69.553  -0.915   0.005   0.336   0.660
B M  3HHRB                139    0.126  25.133  -0.269   0.003   0.718   0.279
E V  3HHRB                140    0.221  33.937   0.461   0.001   0.900   0.099
B L  3HHRB                141    0.035   6.482  -0.390   0.001   0.959   0.040
E E  3HHRB                142    0.245  42.749   1.259   0.001   0.959   0.040
B Y  3HHRB                143    0.032   6.774   0.500   0.001   0.959   0.040
B E  3HHRB                144    0.168  29.280   1.298   0.001   0.959   0.040
B L  3HHRB                145    0.021   3.900   1.251   0.001   0.959   0.040
B Q  3HHRB                146    0.207  36.881   1.479   0.001   0.959   0.040
B Y  3HHRB                147    0.114  24.362   0.882   0.001   0.959   0.040
B K  3HHRB                148    0.262  53.914   1.242   0.001   0.900   0.099
E E  3HHRB                149    0.460  80.362   0.732   0.002   0.816   0.182
E V  3HHRB                150    0.542  83.352   0.507   0.004   0.197   0.799
E N  3HHRB                151    0.735 107.589   0.117   0.005   0.045   0.951
E E  3HHRB                152    0.631 110.288  -0.393   0.005   0.015   0.979
E T  3HHRB                153    0.684  94.940   0.279   0.018   0.047   0.935
E K  3HHRB                154    0.576 118.401   1.340   0.005   0.336   0.660
B W  3HHRB                155    0.140  33.694  -0.077   0.004   0.616   0.381
E K  3HHRB                156    0.469  96.473   0.587   0.001   0.900   0.099
E M  3HHRB                157    0.478  95.648  -0.103   0.001   0.900   0.099
E M  3HHRB                158    0.302  60.510   0.814   0.001   0.900   0.099
E D  3HHRB                159    0.535  77.036  -0.293   0.002   0.816   0.182
E P  3HHRB                160    0.400  56.803  -0.425   0.004   0.616   0.381
E I  3HHRB                161    0.475  87.838  -1.288   0.004   0.420   0.576
E L  3HHRB                162    0.642 117.459  -0.553   0.004   0.197   0.799
E T  3HHRB                163    0.525  72.776  -0.474   0.005   0.336   0.660
E T  3HHRB                164    0.290  40.251  -0.119   0.003   0.718   0.279
E S  3HHRB                165    0.433  50.701   0.632   0.001   0.900   0.099
B V  3HHRB                166    0.088  13.541   0.814   0.001   0.959   0.040
E P  3HHRB                167    0.326  46.316   0.906   0.001   0.900   0.099
B V  3HHRB                168    0.036   5.518  -0.282   0.002   0.816   0.182
E Y  3HHRB                169    0.436  93.088   0.096   0.021   0.451   0.528
E S  3HHRB                170    0.417  48.861  -0.571   0.004   0.138   0.858
B L  3HHRB                171    0.073  13.311  -0.660   0.004   0.085   0.910
E K  3HHRB                172    0.472  97.049   1.143   0.004   0.085   0.910
E V  3HHRB                173    0.497  76.312  -0.138   0.005   0.045   0.951
E D  3HHRB                174    0.620  89.371  -1.342   0.005   0.015   0.979
B K  3HHRB                175    0.185  38.157   0.233   0.005   0.262   0.733
E E  3HHRB                176    0.413  72.238   1.546   0.002   0.816   0.182
B Y  3HHRB                177    0.062  13.292   1.136   0.001   0.959   0.040
B E  3HHRB                178    0.184  32.127   1.919   0.001   0.959   0.040
B V  3HHRB                179    0.015   2.229   1.324   0.001   0.959   0.040
B R  3HHRB                180    0.087  19.877   1.221   0.001   0.959   0.040
B V  3HHRB                181    0.015   2.259   1.484   0.001   0.959   0.040
B R  3HHRB                182    0.106  24.274   1.207   0.001   0.959   0.040
B S  3HHRB                183    0.015   1.758   1.008   0.001   0.900   0.099
B K  3HHRB                184    0.160  32.953   1.530   0.002   0.816   0.182
B Q  3HHRB                185    0.254  45.418  -0.060   0.004   0.514   0.481
E R  3HHRB                186    0.472 108.042  -0.331   0.005   0.045   0.951
E N  3HHRB                187    0.672  98.352  -2.052   0.003   0.003   0.994
E S  3HHRB                188    0.565  66.253  -1.899   0.018   0.019   0.964
E G  3HHRB                189    0.431  33.943  -1.418   0.005   0.015   0.979
E N  3HHRB                190    0.385  56.379   0.288   0.018   0.088   0.893
B Y  3HHRB                191    0.144  30.666  -0.696   0.018   0.088   0.893
B G  3HHRB                192    0.123   9.664  -1.848   0.018   0.047   0.935
E E  3HHRB                193    0.540  94.303  -0.094   0.018   0.047   0.935
B F  3HHRB                194    0.158  31.791  -0.521   0.018   0.047   0.935
B S  3HHRB                195    0.162  18.986  -0.614   0.018   0.047   0.935
E E  3HHRB                196    0.491  85.830  -0.217   0.019   0.141   0.840
E V  3HHRB                197    0.408  62.756  -0.709   0.004   0.420   0.576
B L  3HHRB                198    0.179  32.848   0.840   0.003   0.718   0.279
E Y  3HHRB                199    0.421  89.968   0.478   0.001   0.900   0.099
B V  3HHRB                200    0.120  18.475   0.799   0.001   0.900   0.099
E T  3HHRB                201    0.432  59.905   1.315   0.002   0.816   0.182
B L  3HHRB                202    0.214  39.257   0.784   0.005   0.336   0.660
E P  3HHRB                203    0.381  54.135  -0.479   0.004   0.138   0.858
E Q  3HHRB                204    0.638 113.911   0.134   0.004   0.197   0.799
E M  3HHRB                205    0.479  95.768  -1.074   0.005   0.015   0.979

Explain the output.  Go back.
```

---
